# Secretion Patterns of Leptin: A Key Component in the Regulation of Energy Homeostasis and Its Therapeutic Applications

**DOI:** 10.3390/biom16071031

**Published:** 2026-07-14

**Authors:** Chen Wang, Xianglu Rong

**Affiliations:** Key Laboratory of Glucolipid Metabolic Diseases, Ministry of Education, Guangdong Provincial TCM Key Laboratory for Metabolic Diseases, Guangdong Provincial Research Center of Integration of Traditional Chinese Medicine and Western Medicine in Metabolic Diseases, Guangdong Pharmaceutical University, Guangzhou 510006, China; 2112142102@stu.gdpu.edu.cn

**Keywords:** leptin, energy metabolism, obesity, endocrine regulation

## Abstract

Leptin is the oldest studied adipokine, and its mechanism of action in the regulation of energy metabolism remains a hot topic of current research. In this paper, we systematically review the progress of clinical and basic research on leptin and energy metabolism from 1994 to 2026. It is shown that leptin can regulate the energy metabolism homeostasis through autocrine, paracrine and neurohumoral pathways (e.g., hypothalamic–leptin–melanocortin axis). In addition, the effects of mainstream weight loss strategies such as dietary control, pharmacological interventions and exercise on leptin levels, and their underlying mechanisms were investigated in this paper, with the aim of providing a theoretical basis for the clinical application of leptin in metabolic diseases (e.g., obesity, diabetes mellitus). Future studies need to further clarify the molecular mechanisms of leptin resistance and explore precise intervention strategies based on the leptin signaling pathway.

## 1. Introduction

Energy homeostasis is the state in which an organism maintains a long-term dynamic equilibrium between energy intake and energy expenditure through a series of physiological regulatory mechanisms. Rather than being simple and static, this equilibrium represents a dynamic, finely regulated process. It is a core physiological process for the maintenance of life and health. Governed by the central nervous system (particularly the hypothalamus), it involves a complex network of communication between multiple organs and numerous signalling molecules [1]. Its purpose is to coordinate energy intake, expenditure and storage to maintain long-term stability in body weight and metabolism. Understanding energy homeostasis is crucial for elucidating the pathogenesis of metabolic diseases such as obesity and diabetes and for developing strategies for their prevention and treatment. Among these, leptin—the “satiety signal” produced by adipose tissue—is a key regulatory molecule involved in maintaining energy homeostasis [2].

Leptin, the best-known adipokine, has been the subject of extensive research since its discovery in 1994 [3]. It has been found that leptin plays a role in energy metabolism by suppressing appetite, increasing energy expenditure and maintaining insulin sensitivity [4]. Research indicates that leptin acts as a homeostatic signal (a fat signal) that regulates body weight [5]; for example, mice lacking the leptin gene (ob/ob mice) and humans with congenital leptin deficiency, which is caused by rare genetic mutations in the leptin gene, become severely obese [6]. However, administering leptin to ob/ob mice reduces their food intake and increases their energy expenditure, leading to an energy deficit and weight loss. Furthermore, leptin monotherapy in obese individuals with congenital leptin deficiency can result in sustained weight loss [6,7,8].

Leptin is a cytokine secreted primarily by white adipose tissue in proportion to fat mass; consequently, leptin levels reflect the body’s fat stores [5]. In terms of energy metabolism, leptin binds to leptin receptors to activate downstream signalling molecules, thereby promoting the body’s energy metabolism. It acts primarily on the hypothalamus and, via the peripheral nervous system, regulates the energy-metabolizing organs (adipose tissue, liver, skeletal muscle, etc.) [9]. Adipose tissue also contains a large number of leptin receptors; this finding demonstrates that leptin may also regulate the physiological activity of adipose tissue via an autocrine pathway [10]. However, in a state of obesity, abnormal proliferation of adipose tissue leads to excessive leptin secretion, resulting in reduced sensitivity to leptin and the development of leptin resistance [11,12,13,14]. This causes disturbances in the body’s energy metabolism and insulin resistance, ultimately leading to a series of metabolic syndromes [15].

This review was conducted to analyze how leptin maintains energy homeostasis through different secretion patterns. The role of various therapeutic approaches for energy homeostasis imbalances in the context of leptin mechanisms is discussed, and gaps and limitations in existing evidence are examined. Finally, this review addresses areas for future research and clinical translation.

## 2. The Role of Leptin in Regulating Energy Metabolism in Obesity

Obesity has become a global public health crisis closely related to the occurrence of type 2 diabetes, cardiovascular diseases and various cancers [16]. Leptin is a key hormone secreted by adipose tissue. As a “fat signal” reflecting the body’s energy reserve, it acts on the central nervous system and plays a core role in the regulation of energy homeostasis [3,17].

### 2.1. The Physiological Role of Leptin in Regulating Energy Metabolism

In a healthy state, leptin is the core hormone for maintaining energy balance, which can prevent the occurrence of obesity within a certain range. The hypothalamus is the core brain region where leptin exerts its effects of suppressing food intake and consuming energy. Leptin acts on two key neuronal populations in the arcuate nucleus (ARC) of the hypothalamus, exerting a bidirectional regulatory effect: it activates pro-opiomelanocortin (POMC) neurons to generate satiety signals while inhibiting neurons expressing agouti-related protein (AgRP) and neuropeptide Y (NPY) to reduce the output of hunger signals [18]. This “dual-system” regulatory mode ensures the precise control of leptin over feeding behavior. The signal transduction and transcription activator 3 (STAT3) signalling pathway acts as a transcriptional activator in this process. Studies in mice have shown that when STAT3 in POMC neurons is knocked out, the expression level of POMC mRNA decreases significantly [19]. In addition, brain regions outside the hypothalamus, such as the brainstem, also participate in mediating some metabolic effects of leptin, forming a distributed leptin response network [20].

In addition, leptin can also activate the sympathetic nervous system, promoting fat breakdown and energy mobilization. These peripheral effects synergize with the central appetite-suppressing effect to jointly maintain energy balance [21].

### 2.2. The Pathological State of Leptin Regulating Energy Metabolism

When obesity continues to develop, excessive fat leads to an increase in the level of leptin in the blood (hyperleptinemia). Despite this elevation, leptin’s effects of suppressing appetite and reducing body weight are significantly weakened. This phenomenon is called “leptin resistance” [12,13,14,22]. The mechanism of leptin resistance is very complex. First, at the systemic level, saturation or dysfunction of the leptin transport system across the blood–brain barrier can reduce the amount of leptin entering the central nervous system. Even when the peripheral leptin level is very high, there will be a relative deficiency of ligands reaching the target sites [23]. At the receiving end, although “leptin receptor downregulation” is a classic hypothesis explaining leptin resistance, it has certain limitations when trying to explain human obesity. A 2006 study found that in the post-mortem human hypothalamus, the gene expression level of leptin receptors was not significantly associated with BMI [24]. Therefore, the loss of leptin function receptors (rather than a simple decrease in density) may be the direct cause of obesity. microRNAs (miRNAs) are also involved in precisely regulating the leptin receptor. For example, the extracellular vesicles (EVs) released by adipocytes and the miRNAs they carry are key factors in regulating central leptin sensitivity. Based on their functional characteristics, these adipose EV miRNAs can be divided into two categories—leptin-sensitizing types, which enhance signal transduction by inhibiting the negative feedback factors of LepR signals, and leptin-desensitizing types, which weaken the signals by inhibiting the downstream effector molecules of LepR signals. In a healthy state, adipose EVs are rich in leptin-sensitizing miRNAs. Deficiency in leptin-sensitizing miRNAs is a key factor driving leptin resistance and obesity [25]. Even if leptin successfully binds to its receptor, the core downstream signalling pathway, the JAK2-STAT3 pathway, is often “stuck” in obesity. First, the overactivation of negative feedback regulators such as the cytokine signalling suppressor of growth hormone 3 (SOCS3) directly inhibits the JAK2-STAT3 signalling pathway, creating a “signalling brake” effect [26]. In addition, the abnormal activation of other signalling pathways can also “involve” the leptin signal. For example, increased mammalian target of rapamycin (mTOR) activity can directly damage the phosphorylation level of STAT3 [27].

At the central level, chronic inflammation of the hypothalamus is considered an important factor driving leptin resistance. Obesity-related factors such as a high-fat diet can activate microglia and astrocytes in the hypothalamus, leading to a local inflammatory response and interfering with the normal transmission of leptin signals [28,29].

The role of leptin in obesity reveals the delicate and complex characteristics of the body’s energy regulation system. From the initial discovery of the “hunger hormone” to the current understanding that resistance to leptin is the core obstacle to overcoming obesity, our process of understanding leptin has driven the evolution of obesity treatment strategies.

## 3. Regulation of Leptin Function Mediated by Leptin Receptors

Leptin receptors belong to the class I cytokine receptor family and generate six subtypes (a–f) through alternative splicing. All leptin receptor subtypes are membrane-bound receptor subtypes except for OB-Re. They mediate almost all the biological effects of leptin, from energy metabolism regulation to immune function modulation, through their diverse structures and tissue distributions [30].

Among them, OB-Rb is the only long-form receptor with a complete intracellular signal transduction domain. It is mainly expressed in the hypothalamus, skeletal muscles and immune cells and is the core regulator of appetite, energy metabolic homeostasis and neuroendocrine function [31]. The remaining membrane receptor subtypes have shorter intracellular domains and belong to short-type receptors. They are mainly involved in the transport, endocytosis, and clearance of leptin. Among them, OB-Ra is the most widely distributed subtype [31].

In terms of physiological mechanisms, the membrane-bound leptin receptor itself does not have intrinsic enzyme activity, and its signal transduction depends on coupling with Janus kinase 2 (JAK2) kinase tyrosine. The activated JAK2 phosphorylates specific tyrosine residues in the intracellular segment of OB-Rb, especially Tyr1138, which in turn recruits and activates the STAT3 transcription factor. After dimerization, STAT3 enters the nucleus and initiates the transcription of target genes (such as the POMC gene) [20]. In addition, OB-Rb can activate other signalling pathways, such as PI3K and MAPK/ERK [32]. In contrast, short receptors (such as OB-Ra) lack the tyrosine residues required for binding Box 2 motif (BOX2) and STAT3, so they cannot effectively activate the STAT3 pathway, and they have little signal transduction ability [33].

As the shortest receptor subtype, sOB-R (OB-Re) lacks transmembrane and intracellular domains and exists in the blood in a soluble form [34]. Based on current research, there are two main mechanisms for the generation of sOB-R: alternative splicing and proteolytic shedding. In humans, sOB-R is mainly produced through the proteolytic cleavage of the extracellular domain of the transmembrane leptin receptor by metalloproteinases, a process called “shedding” [35]. Research has confirmed that ADAM10 (a disintegrin and metalloproteinase) is the key enzyme mediating this cleavage process. Lipotoxicity and apoptosis can enhance the cleavage of sOB-R and increase its release through an ADAM10-dependent pathway [36]. The state of the liver, as the main source of circulating sOB-R, can greatly affect the systemic sOB-R level [37]. Functionally, sOB-R’s influence on leptin’s function is not a one-way effect of inhibition or promotion but represents a complex dynamic balance. First, sOB-R can compete with membrane receptors to bind leptin, thereby inhibiting leptin signal transduction, which is particularly evident at high sOB-R levels [38]. Second, sOB-R can delay renal clearance by binding to leptin and prolonging its plasma half-life [39]. Therefore, under normal physiological conditions, sOB-R and leptin form a dynamic buffer system to maintain a stable output of leptin signals.

Research has proven that free leptin exerts the greatest biological effects in the body. All leptin detected in cerebrospinal fluid is in the free form [40], indicating that leptin’s transport across the blood–brain barrier depends on its free state. Importantly, because the binding sites of bound leptin are occupied by sOB-R, it cannot bind to the long-form functional receptor Ob-Rb on the cell membrane. Therefore, it cannot activate the JAK2-STAT3 signalling pathway to exert its usual effects [38].

Under physiological conditions, these free leptin molecules are transported into the brain via the Ob-Ra receptors on the blood–brain barrier. After reaching the ARC of the hypothalamus, they bind to Ob-Rb, activate the JAK2-STAT3 pathway, up-regulate the expression of the anorectic neuropeptide POMC and simultaneously inhibit the orexigenic NPY. Ultimately, this leads to a reduction in food intake and an increase in energy expenditure [41]. Therefore, when in a normal physiological state, leptin can cause “metabolic improvement”. For example, supplementing recombinant leptin can effectively reduce liver weight, fasting blood glucose, and serum insulin levels in ob/ob mice [42]. Higher leptin bioavailability is associated with better preservation of white matter microstructure, and higher leptin bioavailability in middle age may protect against dementia [43].

However, in a state of obesity, adipose tissue proliferates extensively, leading to excessive secretion of leptin and a significant increase in the level of free leptin. Nevertheless, individuals still maintain a high food intake and low energy consumption; that is, they demonstrate “leptin resistance” [6]. In a state of obesity, the pathological functions of leptin, such as “promoting inflammation and causing damage”, may be retained or even amplified. For example, in the liver, the increased free leptin promotes liver inflammation and fibrosis by upregulating the expression of Transforming Growth Factor-beta (TGF-β) [44]. Free leptin is also a key connection between obesity and atherosclerosis. High levels of free leptin damage blood vessels through multiple mechanisms: leptin induces oxidative stress in endothelial cells, promotes the expression of inflammatory factors and impairs the vasodilatory function mediated by nitric oxide (NO) [45]. Leptin can also act as a mitogenic factor, stimulating the proliferation of vascular smooth muscle cells and their migration to the intima and participating in plaque formation [46]. It can enhance platelet aggregation, increase the expression of plasminogen activator inhibitor-1 (PAI-1) and promote thrombus formation [47]. In tumor biology, leptin has been proven to promote the proliferation and survival of various tumor cells [48,49]. In liver cancer, the activation effect of leptin on hepatic stellate cells may promote the evolution of liver cirrhosis to liver cancer [50]. However, some studies have also observed that leptin has immunomodulatory antitumor effects in certain tumors (such as liver cancer) models, suggesting that its effects are tissue-specific [51].

The measurement and interpretation of free leptin levels are becoming important tools for the assessment of metabolic diseases. Compared with total leptin, free leptin more accurately reflects the amount of “effective” leptin that can cross the blood–brain barrier and act on target tissues [40]. Theoretically, it is most ideal to directly and accurately measure the absolute concentration of free leptin. However, such methods often incur high costs, involve complex procedures, take a long time, and have extremely high skill and experimental requirements, making them difficult to popularize in routine clinical or large-scale epidemiological studies. Therefore, the free leptin index (FLI) has become an economical and efficient alternative [52]. The relative proportion of free leptin can be estimated as the ratio of total serum leptin to sOB-R and is widely used to evaluate biological activity or resistance to leptin. However, values measured by different detection methods (ultra-centrifugation, gel filtration chromatography, ELISA, etc.) vary greatly. Moreover, the lack of a unified detection threshold for sOB-R method also affects the accuracy of free leptin calculation, making it difficult to directly compare the values across different studies [53]. In recent years, scientists have also developed the “bioactive leptin (biolep)” detection method—directly using leptin receptor proteins to capture leptin that can bind to them. In essence, it measures free leptin with receptor-binding ability [54]. This detection method is expected to more accurately reflect the functional status of the leptin system, especially in special cases such as functional leptin deficiency.

## 4. Mechanisms Regulating Leptin Secretion and Energy Metabolism Homeostasis

### 4.1. Primary Mode of Secretion—Endocrine

#### 4.1.1. Target Organ—The Hypothalamus

**Figure 1:**
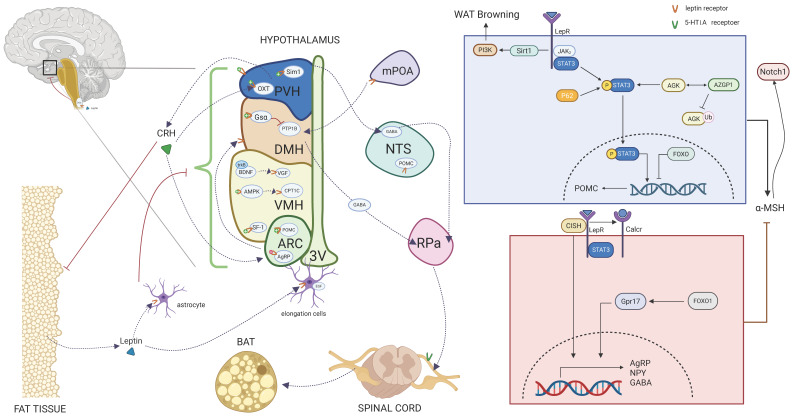
Neuro-humoral regulation. DVC: Dorsal vagal complex; PVH: Paraventricular nucleus of the hypothalamus; DMH: Dorsomedial nucleus of the hypothalamus; VMH: Ventromedial nucleus of the hypothalamus; ARC: Arcuate nucleus of the hypothalamus; 3V: Third ventricle; mPOA: Medial preoptic area; NTS: Nucleus of the solitary tract; RPa: Nucleus Raphe Pallidus; BAT: Brown adipose tissue; CRH: Corticotropin-releasing hormone; Sim1: SIM bHLH transcription factor 1; OXT: Oxytocin; Gsα: Stimulatory G-protein alpha subunit; PTP1B: Protein tyrosine phosphatase 1B; TrkB: Tyrosine receptor kinase B; BDNF: Brain-derived neurotrophic factor; VGF: Neurosecretory protein VGF; AMPK: AMP-activated protein kinase; CPT1C: Carnitine palmitoyltransferase 1C; SF-1: Steroidogenic factor-1; POMC: Pro-opiomelanocortin; AgRP: Agouti-related Protein; GABA: Gamma-aminobutyric acid; PI3K: Phosphoinositide 3-Kinase; SIRT1: Sirtuin 1; LepR: Leptin receptor; JAK2: Janus kinase 2; STAT3: Signal transducer and activator of transcription 3; p62: Sequestosome-1; pSTAT3: Phosphorylated signal transducer and activator of transcription 3; AGK: Acylglycerol kinase; AGK (ub): Ubiquitinated acylglycerol kinase; AZGP1: Zinc-alpha-2-glycoprotein; FOXO: Forkhead box protein O; FOXO1: Forkhead box protein O1; Notch1: Neurogenic locus notch homolog 1; α-MSH: Alpha-melanocyte-stimulating hormone; CISH: Cytokine-inducible SH2-containing protein; Calcr: Calcitonin receptor; Gpr17: G-protein-coupled receptor 17; NPY: Neuropeptide Y. Arrows indicate activation/promotion, while bars (⊥) indicate inhibition. Different colors/shapes are used only to distinguish different components and do not imply functional differences.

The effects of leptin on energy metabolism primarily involve central nervous system signalling pathways [20]. A comparison between hypothalamic leptin receptor knockout mice and db/db mice revealed that the two groups exhibit similar metabolic phenotypes. Although the residual leptin signalling in the hypothalamic leptin receptor knockout mice is still capable of regulating their physiological functions to some extent, this compensatory effect is insufficient to reverse their obese phenotype [55]. This demonstrates that the hypothalamus is the primary site for leptin-mediated regulation of the body’s energy balance. Hypothalamic extensor cells play a key role in this process; for the transcellular transport of leptin, leptin and epidermal growth factor (EGF) must sequentially activate the LepR (leptin receptor):EGFR complex, enabling leptin to traverse the brain and reach its target neurons [56] (Figure 1) (Table 1).

The central melanocortin system plays a key role in the satiating effects of leptin and the activation of the sympathetic nervous system [80]. This system comprises a variety of cell types, including neurons expressing POMC, AgRP, melanocortin-3 receptor (MC3R) and melanocortin-4 receptor (MC4R) [62]. Research has shown that corticotropin-releasing hormone (CRH) promotes the degradation of POMC in the anterior pituitary; it was found that treatment with a CRH antagonist (αCRH) reduces the effect of leptin on the expression of c-fos-like immunoreactivity (cFLI) in the paraventricular nucleus (PVN) and ventromedial hypothalamus (VMH), thereby attenuating leptin’s appetite-suppressing and weight-reducing effects. The study also found a strong negative correlation between CRH and peripheral leptin levels, suggesting that leptin stimulates the hypothalamus to release CRH, which in turn inhibits leptin secretion by fat cells, thereby maintaining a dynamic equilibrium [79]. In the ARC, leptin exerts its appetite-suppressing effect by upregulating the expression of POMC and downregulating the expression of AgRP in neurons [63]. Among these, STAT3 is a transcription factor involved in a variety of biological functions [20]; it acts as a downstream signalling molecule of leptin and serves as an essential “messenger” for transcriptional regulation in both POMC neurons and AGRP/NPY neurons [20]. Other genes in these neurons also play an important role in leptin-mediated energy metabolism. In mice, specific knockout of Sir2-type 1 (Sirt1) in POMC neurons severely impairs the normal binding of leptin to phosphoinositide 3-kinase (PI3K) signalling in POMC neurons, thereby disrupting the remodelling of periglandular white adipose tissue (WAT). At the same time, in mutant mice, sympathetic activity and browning of periglandular fat are also reduced, suggesting that Sirt1 in POMC neurons plays an important physiological role in regulating visceral fat browning [64]. Similarly, through genetic knockout and overexpression in POMC neurons, it was found that the presence of both Sequestosome 1 (p62) and zinc-α2-glycoprotein (AZGP1) genes contribute to increased leptin sensitivity; however, in terms of mechanism, p62 interacts with STAT3, promoting its phosphorylation to initiate POMC transcription and enhance leptin sensitivity. This indicates that p62 can directly regulate STAT3/POMC signalling and enhance leptin sensitivity [73]. AZGP1, in contrast, enhances leptin-JAK2-STAT3 signalling and increases leptin sensitivity by interacting with acylglycerol kinase (AGK) to block its ubiquitin-mediated degradation [58].

Cytokine-induced SH2 domain protein (CISH) is co-expressed with the leptin receptor on AgRP neurons in the ARC; the absence of CISH leads to a reduction in the basal expression of AgRP in the ARC of the brain. Mice lacking CISH are more sensitive to leptin; although CISH expression itself is not regulated by leptin, CISH’s negative regulation of leptin’s anorexigenic effects makes it a key regulator of metabolic balance in vivo [74]. As our understanding of AgRP neurons has increased, it has been discovered that they secrete NPY, AgRP and γ-aminobutyric acid (GABA) simultaneously; they are, therefore, also referred to as NAG neurons. GABAergic RIP-Cre neurons in the ARC can co-ordinately drive energy expenditure, enhance the thermogenic effects of leptin and prevent diet-induced obesity [89]. Neurons in the ARC that express both calcitonin gene-related peptide receptors (Calcr) and leptin receptors include NAG neurons and non-NAG neurons. Experiments have shown that leptin can directly activate LepRb and Calcr in these cells, producing related effects [90]. Mice lacking forkhead box O1 (FOXO1) in POMC or AgRP neurons exhibit favorable metabolic characteristics, characterized by a lean body conformation and increased sensitivity to insulin and leptin. Specific knockout of the G-protein-coupled receptor 17 (Gpr17) gene in AgRP neurons produces the same phenotype as knockout of the FOXO1 gene in the same cell type, providing preliminary evidence that the G-protein-coupled receptor Gpr17 acts as an effector of the appetite-promoting signal from FOXO1 in AgRP neurons [66].

α-Melanocyte-stimulating hormone (α-MSH) is a melanocortin receptor agonist produced by POMC neurons, whereas AgRP is an endogenous melanocortin receptor antagonist [91]. Experiments have shown that α-MSH can enhance leptin sensitivity and preadipocyte proliferation by activating the Neurogenic locus notch homolog 1 (Notch1) signalling pathway whilst simultaneously inhibiting endoplasmic reticulum stress in preadipocytes [78]. The G-protein stimulatory α-subunit (Gsα) facilitates the coupling of various receptors, including MC4R, to intracellular cAMP. Following the generation of Gsα-deficient mice, impaired leptin signalling was observed in the dorsomedial hypothalamus (DMH), accompanied by increased expression of the leptin signalling inhibitor protein tyrosine phosphatase 1B (PTP1B) in the DMH. This may account for the mice’s excessive food intake, reduced energy expenditure, decreased locomotor activity, and diminished cold-induced thermogenesis [75]. Brain-derived neurotrophic factor (BDNF) is one of the endogenous ligands for the tyrosine kinase receptor TrkB, and the VMH is a key site at which BDNF inhibits high-fat diet (HFD)-induced obesity [76]. VGF (VGF nerve growth factor inducible), a propeptide, is particularly abundant in the hypothalamus, and its expression is upregulated by environmental enrichment (EE). Research findings indicate that hypothalamic VGF expression is regulated by leptin, melanocortin receptor agonists and food deprivation and largely parallels BDNF expression [77].

POMC neurons are also found in the nucleus tractus solitarius (NTS) of the medulla oblongata in the brainstem; in recent years, the importance of brainstem nuclei in regulating energy balance has been recognized. The forebrain and hindbrain are connected by extensive synaptic networks, enabling them to act both independently and in concert in regulating the body’s energy metabolism. Research has shown that activating leptin receptors in the NTS of the hindbrain can influence the effects of leptin on the forebrain via neural networks [92,93]. For example, leptin in the posterior brain enhances the precision of energy balance control by lowering the threshold for leptin signalling in the anterior brain [59]. Metabolic activity in the forebrain is transmitted via neural pathways to the hindbrain, thereby influencing peripheral tissues. For example, the thermogenic effect of leptin, mediated by RIP-Cre GABAergic neurons in the ARC, may involve signals from PVH neurons being transmitted via NTS GABAergic neurons to neurons in the raphe pallidus (RPa), which in turn activate 5-HT(1A) receptors on local pre-motor sympathetic neurons, ultimately regulating BAT thermogenesis through neuronal hyperpolarization. Evidence from recent research suggests that Lepr neurons in the dorsal dorsomedial hypothalamic nucleus (dDMH) and docosahexaenoic acid (DHA) integrate signals from upstream Lepr neurons in the preoptic area (POA) and the ARC, releasing equal amounts of the neurotransmitters GABA and glutamate, respectively, which are transmitted downstream to regulate thermoregulation and dynamically adapt to various environmental changes, including ambient temperature and energy status. For example, glutamate is released at the synapse and binds to neurons in the globus pallidus anterior, activating pre-sympathetic motor neurons in the RPa, which in turn stimulate thermogenesis in BAT [89,94]. Furthermore, lepr-positive glial cells in the dorsal vagal complex (DVC) at the posterior end of the brainstem may be involved in the transport of leptin into the brainstem, and the leptin-activated neural circuits in the hypothalamus may access white adipose tissue via the DVC [95].

In addition to the involvement of the melanocortin system, there are other hypothalamus-mediated leptin pathways within the central nervous system [96]. Research has shown that the VMH is involved in leptin’s role in suppressing food intake, suggesting that the VMH also contains LepR [60,61]. Leptin can directly activate Steroidogenic Factor-1 (SF-1)-positive neurons in the VMH, which is a key mechanism underlying leptin’s anti-obesity effects. The absence of SF-1 leads to dysregulation of insulin and leptin homeostasis, resulting in increased food intake and delayed-onset obesity under both normal and high-fat dietary conditions. Furthermore, ablation of SF-1 reduces energy expenditure and physical activity, an effect that is more pronounced in aged mice [97]. Carnitine palmitoyltransferase 1C (CPT1C) in the VMH acts as a downstream regulator of AMP-activated protein kinase (AMPK), which governs BAT thermogenesis. Knocking out CPT1C in mice inhibits BAT thermogenesis and weight loss, demonstrating that CPT1C is essential for leptin-driven BAT thermogenesis [72].

Neurons in the PVH also express leptin receptors, which mediate the anti-obesity and thermogenic effects of leptin. Experiments have shown that microinjection of rAAV-lep into the PVH of diet-induced obese rats reduces energy intake and increases energy expenditure; it normalizes body weight and blood levels of leptin, insulin, free fatty acids and glucose, whilst gastric ghrelin secretion increases over the extended observation period. This indicates that enhancing the response to leptin in the PVH can reverse diet-induced obesity and hyperinsulinaemia and block the central stimulatory effects of elevated endogenous ghrelin on food intake and obesity [98]. Leptin can also target a specific subpopulation of Oxytocin (OXT) neurons within the PVH to reduce body weight. The Single-Hearted Origin 1 gene (Sim1) is highly expressed in the PVH and parts of the amygdala. Studies have shown that specific loss of LepR in Sim1 neurons leads to reductions in both peripheral and core body temperature in mice, decreased energy expenditure at room temperature, and impaired non-shivering thermogenesis in response to cold stimulation. This demonstrates the role of LepR signalling in Sim1 neurons—regulating body weight, core body temperature, and non-shivering thermogenesis [99].

In the nervous system, in addition to neurons, there are large numbers of glial cells, which perform auxiliary functions such as supporting, nourishing, protecting and insulating neurons. Indeed, the central nervous system effects of leptin are also dependent on the support of glial cells. Experiments have shown that knocking out the leptin receptor in astrocytes can prevent morbid obesity induced by a high-fat diet and leptin receptor mutations in neurons, suggesting that the absence of leptin signalling in astrocytes helps to better preserve leptin signalling in neurons. This demonstrates that there is a competitive and negative regulatory relationship between leptin signalling in neurons and that in astrocytes. Astrocyte leptin signalling is a key factor contributing to obesity, hyperleptinaemia, and impaired neuronal leptin signalling [100,101,102].

#### 4.1.2. Target Organ—The Liver

Current research indicates that leptin’s primary effect on the liver is to improve glucose metabolism, such as by enhancing glycogen storage and reducing gluconeogenesis; these may constitute important compensatory mechanisms for the suppression of insulin secretion [103]. Leptin can also temporarily enhance insulin’s effect on glycogen synthesis, a phenomenon associated with the inhibition of phosphorylase A; this effect can be reversed by brief incubation with glucagon [104]. In well-differentiated hepatocellular carcinoma cells, treatment with leptin alone had no effect on the insulin signalling pathway; however, pretreatment with leptin transiently enhanced insulin-induced tyrosine phosphorylation of Insulin Receptor Substrate 1 (IRS-1) and the binding of PI3K to IRS-1 whilst simultaneously inhibiting the tyrosine phosphorylation of Insulin Receptor Substrate 2 (IRS-2) and the binding of PI3K to IRS-2. Leptin can also induce serine phosphorylation of Protein Kinase B (PKB) and glycogen synthase kinase 3, but it is less potent than insulin, and the effects of these hormones are not simply additive [71]. These results indicate that there are complex interactions between leptin and insulin signalling pathways [103].

#### 4.1.3. Target Organ—Muscle

As the primary organ of energy metabolism, skeletal muscle is also directly influenced by leptin [105]. Leptin resistance induced by obesity is clearly manifested in skeletal muscle; the onset of obesity inhibits leptin-stimulated fatty acid oxidation in skeletal muscle [88]. Leptin-stimulated fatty acid oxidation in skeletal muscle can be mediated through pathways involving AMPK and acetyl-CoA carboxylase (ACC); however, this effect is confined to muscle fibres, and the function of this pathway is impaired in the obese state. This suggests that leptin resistance in obesity may be associated with impaired fatty acid oxidation via this pathway [68]. In cellular experiments, treatment of C2C12 myotubes with leptin rapidly induced the expression of acyl-CoA oxidase (ACOX), demonstrating that, in the early stages of overnutrition and prior to the development of leptin resistance, peroxisomes may work in conjunction with mitochondria to clear excess lipids from non-adipose tissues [106]. Furthermore, experiments have identified the N-terminal peptide (17-peptide) of leptin as the primary site responsible for its effects on glucose metabolism and energy homeostasis [107]. The mechanisms determining whether obese patients exhibit impaired leptin-stimulated fatty acid oxidation in skeletal muscle, alongside increased fatty acid uptake and esterification, require further investigation.

### 4.2. Supplementary Mechanism—Autocrine Regulation

**Figure 2:**
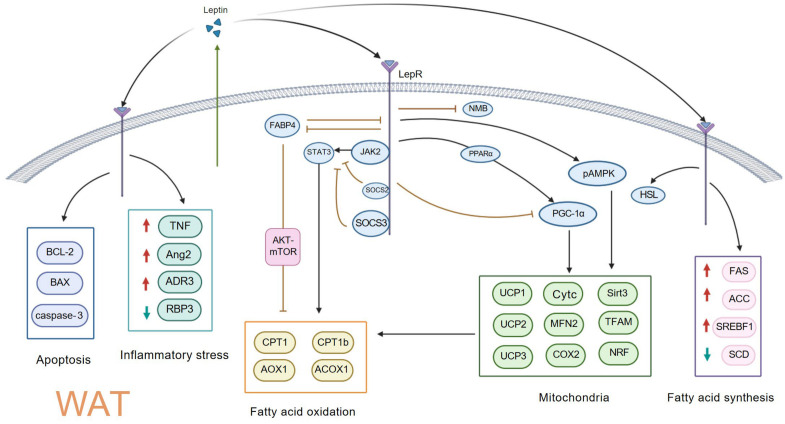
Autocrine regulation. BCL-2: B-cell lymphoma 2; BAX: BCL2-associated X protein; Caspase3: Cysteinyl aspartate specific proteinase 3; TNF: Tumor necrosis factor; Ang2: Angiopoietin 2; ADR3: Adrenergic receptor 3; RBP3: Retinol-binding protein 3; CPT1: Carnitine palmitoyltransferase 1; CPT1b: Carnitine palmitoyltransferase 1B; AOX1: Aldehyde oxidase 1; ACOX1: Acyl-CoA oxidase 1; FAS: Fatty acid synthase; ACC: Acetyl-CoA Carboxylase; SREBP-1: Sterol regulatory element-binding transcription factor 1; SCD: Stearoyl-CoA desaturase; UCP1: Uncoupling protein 1; UCP2: Uncoupling protein 2; UCP3: Uncoupling protein 3; Cyt C: Cytochrome C; MFN2: Mitofusin 2; COX2: Cytochrome c oxidase subunit II; Sirt3: Sirtuin 3; TFAM: Transcription factor A mitochondrial; NRF: Nuclear respiratory factor; FABP4: Fatty acid-binding protein 4; AKT-mTOR: AKT (protein kinase B)—Mechanistic target of rapamycin; STAT3: Signal transducer and activator of transcription 3; JAK2: Janus kinase 2; SOCS2: Suppressor of cytokine signaling 2; SOCS3: Suppressor of cytokine signaling 3; LepR: leptin receptor; NMB: Neuromedin B; PPARα: Peroxisome proliferator-activated receptor alpha; PGC-1α: Peroxisome proliferator-activated receptor gamma coactivator 1-alpha; pAMPK: phosphorylated AMP-activated protein kinase; HSL: Hormone-sensitive lipase; WAT: White adipose tissue. Arrows denote activation, promotion, or secretion, whereas bars (⊥) denote inhibition. Red upward arrows signify an increase, and green downward arrows signify a decrease.

Leptin primarily regulates various signalling molecules and pathways by binding to leptin receptors; it is involved in carbohydrate, lipid and energy metabolism, reduces food intake and increases thermogenesis, thereby promoting weight loss [20]. Adipose tissue serves not only as a secretory organ for leptin but also as its primary target organ. Results from studies involving the knockout of leptin receptors in mouse adipose tissue, as well as in vitro studies of leptin’s effects on rat adipocytes, indicate that adipocytes can regulate energy metabolism in adipose tissue via an autocrine pathway without the involvement of the central nervous system [81,108] (Figure 2).

Early in vitro studies have shown that leptin can directly promote fatty acid oxidation in fat cells, thereby increasing lipid consumption; it can also directly inhibit insulin-mediated fat synthesis in fat cells [109]. Adenovirus-induced leptin overexpression rapidly reduces body fat in rats and promotes the oxidation and breakdown of fatty acids in white adipocytes, leading to the atrophy of these cells, which become filled with mitochondria that are smaller than those found in brown adipocytes. It was found that direct treatment of adipocytes with leptin significantly upregulated the mRNA expression of peroxisome proliferator-activated receptor γ coactivator 1α (PGC-1α), cytochrome c (Cytc), carnitine palmitoyltransferase 1 (CPT1), uncoupling protein 2 (UCP2), and uncoupling protein 3 (UCP3), genes that play a crucial role in fatty acid oxidation and energy expenditure; simultaneously, fatty acid synthase (FAS) expression in adipocytes was reduced, whilst ACC expression and the phosphorylation levels of AMPK were both significantly elevated. These results indicate that leptin stimulates mitochondrial biogenesis and increases fatty acid oxidation, thermogenesis and energy expenditure by activating PGC-1α [110]. Administration of exogenous leptin to ob/ob mice revealed that, following leptin treatment, the levels of hormone-sensitive lipase (HSL), UCP2, adrenergic receptor 3 (ADR3), mitofusion protein 2 (Mfn2), sirtuin 3 (Sirt3), sterol regulatory element-binding factor 1 (SREBF1), B-cell lymphoma 2 (Bcl-2), Bcl-2-associated X protein (Bax), cysteine-aspartate protease 3 (Caspase 3), tumor necrosis factor α (TNF-α), adiponectin, and angiopoietin 2 (Ang-2) increased, whilst the expression of stearoyl-CoA desaturase 1 (SCD1), FAS, and retinol-binding protein 4 (RBP4) was reduced. These results indicate that leptin treatment in ob/ob mice alters gene expression in adipose tissue, which not only promotes lipid mobilization and energy expenditure but also contributes to apoptosis and angiogenesis [111].

SOCS3 is considered a leptin-resistant factor; in obese rats fed a high-fat diet, the mRNA and protein levels of SOCS3 in epididymal fat were significantly increased. In vitro experiments revealed that after incubating SOCS3-knockdown adipocytes with 50 nM of leptin for 6 h, the mRNA expression of acetyl-CoA carboxylase (a marker of de novo fatty acid synthesis) was reduced, whilst the expression of acetyl-CoA oxidase mRNA (a marker of fat oxidation) increased, demonstrating that SOCS3 acts as a negative regulator of leptin-mediated fatty acid oxidation in adipocytes [67]. suppressor of cytokine signaling 2 (SOCS2) is another member of the SOCS family and is widely expressed in muscle, nerve, pancreatic and adipose tissues. Studies have shown that leptin increases SOCS2 mRNA levels in mouse inguinal adipose tissue; however, it has been observed that genes associated with fatty acid oxidation that are up-regulated by leptin, such as PGC-1α, nuclear respiratory factor 1 (NRF-1), transcription factor A, mitochondrial (TFAM), carnitine palmitoyltransferase 1B (CPT-1b), aldehyde oxidase 1 (AOX1), cytochrome c oxidase subunit 2 (COX2), and UCP2 are attenuated by SOCS2. Furthermore, SOCS2 reduces the levels of mitochondrial complexes I and III, the fatty acid oxidase Medium-Chain Acyl-CoA Dehydrogenase (MCAD), long-chain acyl-CoA dehydrogenase (LCAD), and cytochrome C, as well as the release of free fatty acids. This indicates that SOCS2 exerts a negative effect on mitochondrial fatty acid oxidation; these effects are closely linked to the LepR/JAK2/AMPK pathway [57].

Neuromodulatory protein B (NMB) is a member of the iridin-like peptide family and has been shown to reduce food intake when administered systemically. Its expression in adipose tissue is regulated by leptin; following leptin administration to both obese and control mice, mRNA expression of NMB in adipose tissue was reduced in both groups. Given its anorexigenic effects, it may represent a novel physiological mechanism for regulating appetite via the “adipose–hypothalamic axis” [83]. Activation of fatty acid-binding protein 4 (FABP4) reduces the expression of leptin, CPT1 and AOX1 in mouse adipocytes; conversely, leptin treatment downregulates FABP4. FABP4 can reverse leptin-induced mitochondrial fatty acid oxidation, and this effect is closely associated with the inhibition of the Akt/mTOR signalling pathway [82].

## 5. The Circadian Rhythm of Leptin Secretion

Research has found that there is a significant 24 h rhythm in leptin secretion. For example, in healthy people, leptin secretion shows a pattern of being high at night and low during the day, reaching a peak from late at night to early morning (around 00:00–02:30) and dropping to a trough in the afternoon [112]. However, multiple experiments on humans and animals have shown that changes in circulating leptin levels are affected primarily by eating, and a high-fat diet can significantly alter the circadian rhythm of leptin levels [113,114]. Adjusting one’s eating time according to their circadian rhythm can significantly improve the metabolic function of leptin [115].

In terms of central regulation, the circadian rhythm precisely regulates sleep through the suprachiasmatic nucleus (SCN) [116]. In a test of 58 healthy individuals, it was found that during sleep, the higher the proportion of rapid eye movement sleep, the less leptin is secreted [117]. In addition, during parts of the circadian rhythm, the SCN sends signals to prompt the pineal gland to secrete melatonin, which makes people feel sleepy [116]. After the pineal gland was removed from rats, they exhibited manifestations related to leptin resistance. Subsequently, when these pinealectomized rats were treated with melatonin, their leptin sensitivity was significantly restored, which proves that there is an interaction between melatonin and leptin in the hypothalamus [118]. In mice lacking the Brain and Muscle ARNT-Like 1 (BMAL1) gene in leptin receptor neurons, the preference for a high-fat diet is reduced, and they gain less weight at first. This mechanism can regulate the pattern of hedonic eating and affect metabolic homeostasis. It reveals the influence of the rhythm–leptin loop on metabolism [119]. When REV-ERBα and REV-ERBβ, the “brake” genes of the circadian rhythm in the mouse hypothalamus, are damaged, the sensitivity to damage is reduced, indicating a correlation between regulation of the circadian rhythm and leptin metabolism [85].

In addition, the coupling of the central and peripheral clocks controls the endocrine feedback balance of leptin. For example, the activation of the fat-specific succinate receptor 1 (SUCNR1) can control leptin expression in an AMPK/JNK-C/EBPα-dependent manner through the circadian clock [86]. All of the above studies indicate that the circadian rhythm system is deeply involved in the secretion regulation and metabolic function of leptin.

## 6. Therapeutic Transformation

### 6.1. Medicines

Some obese patients exhibit leptin resistance; consequently, leptin sensitizers, rather than leptin itself, are expected to serve as therapeutic agents for obesity. Leptin sensitizers are primarily intended to address conditions characterized by excessively high leptin concentrations or leptin resistance. Studies have shown that oral administration of 1,3-butanediol (BD) has a therapeutic effect on obese mice with leptin resistance; this effect is closely associated with increased ATP concentrations in the hypothalamus and elevated plasma levels of β-hydroxybutyrate [120]. Inhibitors of the cellular histone deacetylase 6 (HDAC6), such as tolvaptatin A, are also potent leptin sensitizers and anti-obesity agents. Mechanistically, they confer central leptin sensitivity by inhibiting peripheral HDAC6. Notably, the anti-obesity effects of tolvaptatin A are attenuated in animals accompanied by a reduction in the central leptin-melanocortin feedback loop, including in db/db and MC4R-KO mice [87]. Resveratrol also reduces leptin secretion from fat cells in rats [121]. Rapamycin, an inhibitor of the mTOR, reduces food intake and fat mass in diet-induced obese mice but is ineffective in animals with leptin deficiency. Bowen Tan recently discovered that increased mTOR activity in POMC neurons leads to leptin resistance, whilst rapamycin restores the action of leptin on POMC neurons. This suggests that rapamycin can suppress enhanced mTOR activity, thereby improving leptin resistance and alleviating associated symptoms of obesity [122].

Obesity patients often experience a ‘weight loss plateau’ whilst trying to lose weight, and the advent of leptin sensitizers may well break the vicious cycle of ‘weight loss followed by weight regain’. This is because when a person loses weight, the number of fat cells decreases and the secretion of leptin falls accordingly; upon receiving this signal, the brain activates a compensatory mechanism by increasing appetite and reducing metabolic rate [84]. Leptin sensitizers not only restore the brain’s sensitivity to high concentrations of leptin during periods of obesity characterized by leptin resistance—thereby directly suppressing appetite, boosting metabolism and promoting weight loss. However, once weight has been lost and leptin levels have returned to the normal range, they can help maintain weight and prevent weight regain by ensuring the brain remains sensitive to these normal leptin levels. Most currently available leptin sensitizers are effective only in cases of high leptin concentrations and leptin resistance and do not produce any significant response in individuals with normal leptin levels [87]. Consequently, future research could explore approaches that go beyond the sole use of leptin sensitizers, such as integrating them into a comprehensive treatment regimen—for example, by combining them with other types of medication—or utilizing them as a chronic disease management medication for long-term use to regulate energy balance and maintain normal physiological states. However, even though many similar drug candidates have shown promising results in laboratory studies, there is still a long journey from animal trials to the successful development of drugs that are both effective and safe for human use. At present, there is no “leptin sensitizer”, in the strict sense of the term, that has been widely approved for clinical use. Some existing drugs (such as the glucagon-like peptide-1 (GLP-1) receptor agonists semaglutide and tirzopentide) have been found to potentially improve leptin sensitivity indirectly, in addition to their primary functions, but they are not dedicated leptin sensitizers [65].

In addition to leptin sensitizers, there are also drugs that exert leptin-related anti-obesity effects by activating downstream leptin pathways. Konjac glucomannan (KGM) significantly reduced leptin and fatty acid signalling in adipose tissue, activated thermogenesis in brown adipose tissue, suppressed the expression of POMC and activated the expression of AgRP, thereby reducing food intake and increasing energy expenditure [123]. Studies have also shown that Panax notoginseng saponins (PNSs) can participate in the remodelling of brown adipose tissue by regulating the leptin/AMPK/STAT3 signalling pathway induced by the gut microbiota. This leads to increased energy expenditure and a reduction in obesity [69].

### 6.2. Diet

Calorie restriction significantly reduces the concentration of leptin in the blood [124,125,126,127]. In addition, studies have shown that this reduction in leptin is accompanied by an increase in soluble leptin receptors, which has been attributed primarily to the shedding of plasma membrane ObRa receptors from white adipose tissue, rather than to increased ObRe expression [128]. This is a normal physiological response of the body to adapt to the state of energy shortage. Its core purpose is to conserve energy and promote eating, but this effect weakens over time. Over a four-week period of monitoring 21 obese women, it was found that the average leptin level decreased significantly by 66% in the first week of fasting, and then the proportion gradually decreased. This shows that in obese patients, short-term changes in energy intake are the main factor regulating plasma leptin concentration. The leptin concentration is related to blood glucose changes and can overcome the regulatory influence of fat mass [129]. A meta-analysis that included 16 RCTs (593 participants) found that fasting regimens such as intermittent fasting, time-restricted feeding (TRF), and alternate-day fasting (ADF) could significantly reduce leptin levels. The study also found that the effects were more significant when the intervention duration was ≥12 weeks [130]. Therefore, fasting can lead to weight loss, but after resuming free eating, the weight will quickly rebound [131]. Comparing caloric restriction and hypothalamic leptin gene therapy, rats under caloric restriction had a higher mass of abdominal white adipose tissue, higher levels of serum leptin and adiponectin, and a higher bone marrow fat content [132]. In a 2017 study, it was found that both energy expenditure caused by acute food deprivation and excessive energy storage caused by high-fat diet feeding can weaken the effect of leptin on inhibiting excitatory synaptic input to the neurons in the lateral hypothalamic area (LHA) that express orexin and melanin and project to the ventral tegmental area (VTA) [133]. This may help explain the manifestations of leptin resistance that occur during fasting for weight loss. Therefore, a meta-analysis including 87 RCTs shows that dietary restriction combined with exercise is most effective at reducing leptin levels [134].

The study also found that dietary patterns can affect the metabolic function of leptin. For, example although there are some limitations, studies on animals and humans have shown that a diet high in fat, carbohydrates, fructose, and sucrose, as well as low in protein, is a factor leading to leptin resistance [124]. However, a 2021 study found that increasing dietary sucrose intake leads to leptin resistance, and this mechanism is related to differences in specific parts of leptin signalling in the hypothalamus [135]. Further research shows that exercise training combined with a plant-based diet can significantly reduce leptin levels [136]. Excessive intake of saturated fatty acids can lead to the occurrence of leptin resistance. For example, coconut oil, which has saturated fatty acids as its main component, can induce resistance to leptin and insulin in the hypothalamus and peripheral tissues of healthy mice [137]. Cross-sectional studies have found that the Mediterranean diet, characterized by a low intake of saturated fatty acids (7–8%) and a relatively high intake of unsaturated fatty acids, is likely to affect the body mass index and maximal oxygen uptake through the mediating effect of leptin [138]. In addition, a meta-analysis of 48 experiments found that a simple low-fat diet had no significant effect on leptin levels [139].

### 6.3. Exercise

Traditionally, it has been believed that weight loss through exercise is achieved primarily by burning calories. However, research suggests that exercise itself acts as a powerful biological signal that directly regulates the levels and sensitivity of the key hormone leptin, thereby improving obesity at both the hormonal and neural levels. It is not merely a matter of “burning calories”, but rather of “resetting” the body’s energy regulation system.

For example, exercise can lower leptin levels. Following exercise, leptin concentrations in the blood decrease (both at the mRNA level and in plasma), but this effect is delayed; this suggests that exercise does not reduce the leptin gene itself but rather regulates its “expression” process [140,141,142]. Research has found that a decrease in blood leptin levels can be observed within a few hours of acute exercise (a single session), whilst there is no corresponding change in fat mass. This suggests that exercise directly triggers a physiological mechanism that instructs fat cells to “reduce the synthesis and secretion of leptin” rather than relying on a reduction in fat cell size to decrease secretion [2]. This rapid decline in leptin concentration, independent of body fat mass, is likely an adaptive response by the body; it temporarily reduces “satiety signals”, helping to ensure that individuals retain sufficient appetite after exercise to consume the energy and nutrients needed to repair the body. This suggests that every bout of exercise offers immediate metabolic benefits [141]. The likely explanation for this mechanism is that a single bout of exercise immediately enhances the hypothalamus’s response to leptin signals (such as increased extracellular signal-regulated kinase 1/2 (ERK1/2) phosphorylation), thereby boosting energy expenditure [70]. Following long-term exercise training, however, overall leptin levels decline; this is partly due to a reduction in total body fat, which indirectly affects leptin production. More importantly, the brain has become more sensitive to leptin; even though total leptin levels have fallen, the brain’s response to it has become stronger (this is why exercise has a more pronounced effect on people with normal metabolism, as their leptin system is inherently more sensitive) [143]. Furthermore, the experiments revealed that when the GP130 protein was knocked out in mice, the weight-loss effects of exercise were significantly reduced, suggesting that exercise relies on Interleukin-6 (IL-6) signals released by fat cells to enhance the hypothalamus’s sensitivity to leptin and insulin. This demonstrates that the effects of exercise are not mediated by a single hormone but rather result from the synergistic interaction of multiple hormones and cytokines—including leptin, insulin and IL-6—at the network level [144].

Furthermore, obesity is often accompanied by chronic inflammation, which interferes with leptin signalling; exercise can suppress inflammation and thus help restore the function of the leptin signalling pathway. However, research has shown that it is moderate-intensity exercise that effectively improves leptin resistance and inflammation, whereas high-intensity exercise may trigger a strong inflammatory response, which could actually offset the benefits of exercise in improving leptin resistance [145].

It is well known that exercise and controlling food intake are key strategies for managing obesity. For over a decade, scientists have been investigating whether leptin plays a role in the effects of exercise on obesity. Research on exercise interventions indicates that dietary control can reduce body weight and leptin levels, but only when combined with exercise can it effectively improve leptin resistance and achieve long-term weight management [134,146,147]. Otherwise, at low leptin levels, the body is more prone to feeling hungry and entering an energy-saving mode (a plateau or weight regain). Furthermore, for obese individuals, starting with moderate-intensity aerobic exercise may be an effective and lower-risk method for improving leptin sensitivity and suppressing inflammation [148].

## 7. Conclusions

Leptin, as the earliest identified adipokine, holds a well-established central role in the regulation of energy metabolism. Since the cloning of the leptin gene in 1994, its mechanistic actions within the hypothalamic–leptin–melanocortin axis have been progressively elucidated. Leptin modulates feeding behavior and energy expenditure by activating POMC neurons and inhibiting AgRP/NPY neurons in the hypothalamic arcuate nucleus, primarily through theJAK2/STAT3 signaling pathway. Additionally, leptin receptor signaling in distinct hypothalamic nuclei—including the ventromedial, dorsomedial, and paraventricular nuclei, as well as the nucleus tractus solitarius—cooperatively participates in the regulation of sympathetic outflow, thermogenesis, and glucose homeostasis.

Despite its fundamental physiological significance, the clinical application of leptin-based therapies is severely constrained by leptin resistance, a hallmark of obesity. Accumulating evidence has revealed that leptin resistance arises from multifaceted mechanisms, including excessive negative feedback signaling mediated by SOCS3 and PTP1B, hypothalamic inflammation, modulation of leptin bioavailability by soluble leptin receptors, and the regulatory roles of astrocytes and microglia within the inflammatory microenvironment. In recent years, the identification of novel molecular targets—such as HDAC6 inhibitors, REV-ERB nuclear receptor modulators, YAP/TAZ signaling, and the SUCNR1 pathway—has offered new avenues for restoring leptin sensitivity.

At the clinical translational level, dietary restriction, exercise intervention, and pharmacotherapy remain the primary strategies for improving leptin sensitivity. Caloric restriction effectively reduces circulating leptin levels but concomitantly triggers metabolic adaptation, increasing the risk of weight regain. Exercise training not only modulates leptin levels but also enhances the activation of hypothalamic leptin signaling pathways, with efficacy influenced by exercise intensity, modality, and energy status. Furthermore, emerging strategies including time-restricted eating, specific dietary patterns, and GLP-1 receptor agonists have demonstrated potential in modulating leptin signaling and improving metabolic outcomes.

In summary, leptin orchestrates energy homeostasis through a multilayered central and peripheral regulatory network, and leptin resistance constitutes a core pathophysiological mechanism underlying obesity and related metabolic disorders. Future research should prioritize the following directions: (1) deciphering the cell-type-specific and region-specific functions of leptin signaling across distinct brain nuclei and cell populations—including neurons, astrocytes, microglia, and tanycytes—in the maintenance of energy homeostasis; (2) elucidating the regulatory roles of circadian rhythms, gut microbiota, and epigenetic modifications in leptin sensitivity; (3) developing selective sensitization strategies targeting molecular nodes within leptin signaling pathways, aiming to restore endogenous leptin function while mitigating potential oncogenic risks; and (4) advancing individualized precision intervention paradigms that integrate dietary, exercise, and pharmacological combination therapies for the effective prevention and management of obesity and metabolic diseases.

## 8. Summary and Outlook

Obesity has long been a major challenge in the field of healthcare, and finding solutions to this problem has become a key area of research within the scientific community. Energy metabolism encompasses both energy intake and energy expenditure and ensuring that energy expenditure exceeds energy intake has long been regarded as the primary method of addressing obesity. As the first adipokine to be discovered, leptin has been extensively studied for its role in reducing food intake and increasing energy expenditure. Although some progress has been made in understanding the effects of leptin on metabolic function and its underlying molecular mechanisms, much work remains before it can be applied as a therapeutic agent in clinical practice. Elucidating the network pathways through which leptin exerts its effects will facilitate the exploration of its immense potential in the treatment of metabolic diseases. In summary, understanding the role of leptin in energy metabolism helps uncover new approaches for increasing energy expenditure and reducing energy intake, which is of great significance for the research, prevention and treatment of fat accumulation associated with metabolic disorders.

## Figures and Tables

**Table 1:** Key molecules involved in leptin signaling pathways and their downstream effects.

Signaling Pathway-Related Factors	Mechanism	Object	Reference Number
LepR (Ob-Rb)	Leptin receptor mediates leptin signal transduction and activates the JAK2/STAT3 pathway.	Mouse, Rat, Human	[34,35,36]
JAK2	After binding to LepR, it is phosphorylated and activated, initiating the downstream signal cascade reaction.	Mouse, Cell line	[32,57]
STAT3	Activated by JAK2 phosphorylation, it enters the nucleus to regulate the transcription of target genes such as POMC and mediates feeding inhibition and energy consumption.	Mouse, Rat, Human	[2,33,58,59,60,61]
POMC	After being activated by STAT3, it promotes transcription, generates α-MSH, inhibits food intake, and increases energy consumption.	Mouse, Rat, Human	[41,58,62,63,64,65]
AgRP/NPY	Inhibited by leptin, it promotes food intake; after inhibition, it reduces appetite.	Mouse, Rat	[41,63,66]
SOCS3	Induced by leptin for expression, it negatively feedback inhibits the JAK2/STAT3 signaling, leading to leptin resistance.	Mouse, Rat, Cell line	[27,32,67]
PTP1B	Dephosphorylation of JAK2 negatively regulates leptin signaling and promotes leptin resistance.	Mouse, Cell line	[32]
AMPK	Activated by leptin, it promotes fatty acid oxidation and regulates energy metabolism.	Mouse, Skeletal muscle cells, Adipocyte	[57,68,69]
ACC	Inhibited by AMPK phosphorylation, reducing fatty acid synthesis.	Mouse, Skeletal muscle cells	[68]
Erk1/2 (MAPK)	Activated by leptin, it regulates cell proliferation, differentiation, and energy metabolism; exercise can enhance its phosphorylation in the hypothalamus.	Mouse	[70]
PI3K	Activated by leptin, it participates in the interactive regulation of insulin signaling and regulates glucose metabolism.	Cell line, Rat	[32,71]
mTOR	Regulated by leptin, it participates in energy sensing and feeding regulation.	Mouse	[32]
CPT1C	It is expressed in the ventromedial nucleus of the hypothalamus, participates in the activation of brown fat thermogenesis, and is regulated by leptin.	Mouse	[72]
SIRT1	It is expressed in POMC neurons and is crucial for the homeostatic defense required to resist diet-induced obesity.	Mouse	[64]
p62 (SQSTM1)	The hypothalamus regulates leptin signals, and p62 deficiency leads to leptin resistance and hyperphagia.	Mouse	[19,73]
AZGP1	Regulation of energy homeostasis through leptin-mediated STAT3 phosphorylation in POMC neurons.	Mouse	[58]
CISH	Cytokine-inducible SH,2-containing protein regulating fat accumulation, food intake, and glucose metabolism.	Mouse	[74]
Gpr17	It is expressed in AgRP neurons and regulates food intake as well as the sensitivity to insulin and leptin.	Mouse	[66]
Gsα	Defects in the dorsomedial hypothalamic nucleus lead to obesity, hyperphagia, and reduced thermogenesis, which are associated with impaired leptin signaling.	Mouse	[75]
BDNF	Chronic administration in the ventromedial hypothalamic nucleus can reduce high-fat diet-induced obesity.	Rat	[76]
VGF	Hypothalamic VGF is involved in energy balance and metabolic adaptation.	Mouse	[77]
MC4R	The receptor of α-MSH mediates anorexia and thermogenic effects and is a key effector molecule downstream of leptin.	Mouse, Rat	[62,63]
α-MSH	It is produced by the processing of POMC and acts on MC4R to inhibit food intake and promote thermogenesis.	Mouse	[62,63,78]
CRH	Regulated by leptin, it participates in the hypothalamic–pituitary–adrenal axis and regulates energy balance.	Rat	[79]
TRH	Downregulation of leptin action affects the hypothalamic–pituitary–thyroid axis.	Rat adipocytes	[80]
UCP2	Upregulated by leptin; regulate mitochondrial function and reactive oxygen species; expressed in white adipose tissue.	Mouse adipocytes	[81]
FABP4	Regulation of mitochondrial fatty acid oxidation by leptin in an inverse manner in adipocytes.	Mouse adipocytes	[82]
SOCS2	Inhibit mitochondrial fatty acid oxidation in adipocytes by suppressing the LepR/JAK2/AMPK signaling pathway.	Mouse adipocytes	[57]
Neuromedin B	Expressed in adipose tissue and regulated by changes in energy balance.	Mouse	[83]
Leptin (OB gene)	Adipocytes secrete substances that, as adipose signals, reflect energy reserves. They act on the hypothalamus to inhibit food intake and promote heat production.	Mouse, Rat, Human	[1,3,42,84]
sOB-R	Produced by the proteolytic cleavage of membrane-bound receptor proteins, it regulates the circulating leptin level and bioavailability.	Human, Rat, Cell line	[35,36,37,38,39]
EGFR	Forms shuttles with LepR in ependymal cells to control leptin entry into the brain and lipid metabolism.	Mouse	[56]
MyD88	Involved in high-fat diet-induced hypothalamic inflammation in astrocytes.	Mouse	[28]
TLR4	Mediate the inflammatory response in the hypothalamus and participate in the occurrence of leptin resistance.	Mouse, Microglia	[29]
NF-κB	Hypothalamic inflammatory signals, activated by saturated fatty acids, lead to leptin resistance.	Mouse, Cell line	[28,29]
YAP/TAZ	The effector molecules of the Hippo signaling pathway coordinate fat plasticity and energy balance, decoupling leptin expression from fat mass.	Mouse	[42]
REV-ERB	Hypothalamic nuclear receptors control circadian food intake and leptin sensitivity.	Mouse	[85]
SUCNR1	Signal transduction in adipocytes controls energy metabolism by regulating the biological clock and leptin expression.	Mouse adipocytes	[86]
HDAC6	Inhibiting HDAC6 can restore leptin sensitivity and reduce obesity.	Mouse	[87]
CHOP	Endoplasmic reticulum stress markers regulate the level of soluble leptin receptor through CB1R and promote hepatic leptin resistance.	Mouse, Cell line	[37]
CB1R	Cannabinoid receptor 1 regulates the level of soluble leptin receptor and participates in hepatic leptin resistance.	Mouse, Cell line	[37]
AMPKα	Notoginsenosides promote thermogenesis and beige fat remodeling through the leptin-mediated AMPKα/STAT3 signaling pathway.	Mouse	[69]
CPT1	Leptin promotes CPT-dependent1 fatty acid transport in fatty acid oxidation by activating AMPK.	Mouse, Skeletal muscle, Adipose tissue	[57,68,88]
Notch1	α-MSH promotes preadipocyte proliferation by alleviating endoplasmic reticulum stress-induced leptin resistance and activating the Notch1 signaling pathway.	Mouse	[78]
HIF-1α	Regulation of mitochondrial metabolism by autophagy during leptin-induced cell migration.	Cell line	[48]

LepR (Ob-Rb): Long-form leptin receptor; JAK2: Janus kinase 2; STAT3: Signal transducer and activator of transcription 3; POMC: Pro-opiomelanocortin; AgRP/NPY: Agouti-related protein/neuropeptide Y; SOCS3: Suppressor of cytokine signaling 3; PTP1B: Protein tyrosinepPhosphatase 1B; AMPK: AMP-activated protein kinase; ACC: Acetyl-CoA carboxylase; Erk1/2 (MAPK): Extracellular signal-regulated kinase 1/2 (mitogen-activated protein kinase); PI3K: Phosphoinositide 3-kinase; mTOR: Mammalian target of rapamycin; CPT1C: Carnitine palmitoyltransferase 1C; SIRT1: Sirtuin 1; p62 (SQSTM1): Ubiquitin-binding protein p62 (Sequestosome 1); AZGP1: Zinc-alpha-2-glycoprotein; CISH: Cytokine-inducible SH2-containing protein; Gpr17: G-protein-coupled receptor 17; Gsα: Stimulatory G-protein alpha subunit; BDNF: Brain-derived neurotrophic factor; VGF: Neurosecretory protein VGF; MC4R: Melanocortin 4 receptor; α-MSH: alpha-melanocyte-stimulating hormone; CRH: Corticotropin-releasing hormone; TRH: Thyrotropin-releasing hormone; UCP2: Uncoupling protein 2; FABP4: Fatty acid-binding protein 4; SOCS2: Suppressor of Cytokine Signaling 2; sOB-R: soluble leptin receptor; EGFR: Epidermal Growth Factor Receptor; MyD88: Myeloid differentiation primary response gene 88; TLR4: Toll-like receptor 4; NF-κB: Nuclear factor kappa-B; YAP/TAZ: Yes-associated protein/Transcriptional coactivator with PDZ-binding motif; REV-ERB: Nuclear Receptor Subfamily 1, Group D, Member 1–Member 2; SUCNR1: Succinate receptor 1; HDAC6: Histone Deacetylase 6; CHOP: C/EBP homologous protein; CB1R: Cannabinoid receptor 1; AMPKα: AMP-activated protein kinase catalytic subunit alpha; CPT1: Carnitine palmitoyltransferase 1; Notch1: Notch homolog 1; HIF-1α: Hypoxia-inducible factor-1 alpha.

## Data Availability

No new data were created or analyzed in this study. Data sharing is not applicable to this article.
